# Double Heterozygosity for *BRCA1* Pathogenic Variant and *BRCA2* Polymorphic Stop Codon K3326X: A Case Report in a Southern Italian Family

**DOI:** 10.3390/ijms19010285

**Published:** 2018-01-18

**Authors:** Raffaele Palmirotta, Domenica Lovero, Luigia Stefania Stucci, Erica Silvestris, Davide Quaresmini, Angela Cardascia, Franco Silvestris

**Affiliations:** Department of Biomedical Sciences and Human Oncology, University of Bari Aldo Moro, 70124 Bari, Italy; dom.lovero@gmail.com (D.L.); stuccistefania@gmail.com (L.S.S.); ericasilvestris85@gmail.com (E.S.); davide.quaresmini@hotmail.it (D.Q.); angelcard@hotmail.it (A.C.); francesco.silvestris@uniba.it (F.S.)

**Keywords:** *BRCA1*/*BRCA2*, mutational analysis, double heterozygosity, age of onset, K3326X

## Abstract

Here, we describe a patient with bilateral breast cancer and melanoma, and with a concomitant double variant, namely p.Gln563Ter in *BRCA1* and p.Lys3326Ter in *BRCA2*. The *BRCA2* p.Lys3326Ter (K3326X) (rs11571833) mutation identified in our patient is a debated substitution of thymidine for adenine which is currently regarded as benign polymorphism in main gene databases. Recent studies, however, describe this variant as associated with breast and ovarian tumors. Based on the observation of the cancer’s earliest age of onset in this subject, our purpose was to reevaluate this variant according to recent papers indicating a role of powerful modifier of the genetic penetrance. Genetic testing was performed in all consenting patient’s relatives, and in the collection of the clinical data particular attention was paid to the age of onset of the neoplasia. Following our observation that the our patient with double heterozygosis had an early age of onset for cancer similar to a few rare cases of double mutation for *BRCA1* and *BRCA2*, we also performed an extensive review of the literature relative to patients carrying a double heterozygosity for both genes. In line with previous studies relative to the rare double heterozygosity in both *BRCA1*/*2* genes, we found the earlier onset of breast cancer in our patient with both *BRCA1*/*2* mutations with respect to other relatives carrying the single *BRCA1* mutation. The presence of the second K3326X variant in our case induces a phenotype characterized by early onset of the neoplasia in a manner similar to the other cases of double heterozygosity previously described. Therefore, we suggest that during the genetic counseling, it should be recommendable to evaluate the presence of the K3326X variant in association with other pathogenic mutations.

## 1. Introduction

Both *BRCA1* and *BRCA2* are oncosuppressor genes involved in DNA repair and are commonly mutated in a number of cancers. The identification of molecular mechanisms in inherited diseases as hereditary breast and ovarian cancer syndrome (HBOC) has emphasized the role of these genes in cancer, allowing subjects at risk of cancer to be screened for prevention and, hopefully, early diagnosis [1].

Although mostly somatically mutated, in a relevant number of subjects these genes are expressed with germline mutations that, however, recur within the general population with a prevalence variable equal to 1/400 up to 1/800 individuals [1,2,3]. Several ethnic groups carrying constitutional mutations show significantly higher prevalence, such as the Ashkenazi Jewish with increased prevalence equal to 1/40 subjects of 185delAG and 5382insC and 617delT founder mutations in both *BRCA1*/*2* genes [1]. For the same reasons, the presence of double mutations of *BRCA1* and *BRCA2* is extremely rare in the general population, although not exceptional in Ashkenazi breast-cancer patients [4]. Compared to the huge amount of mutational analysis data for *BRCA1* and *BRCA2* genes, only a few studies report dual mutations in non-Ashkenazi subjects, mostly consisting in the description of a single or of a small number of families [4,5,6,7,8,9].

In the present study, we describe a case of coexisting *BRCA1* p.Gln563Ter and *BRCA2* p.Lys3326Ter double variant in a southern Italian family. The *BRCA2* variant, also known as K3326X, was firstly interpreted as pathogenic, but its identification in control populations with no increase in breast or ovarian cancer occurrence led to its reinterpretation of benign polymorphism [10]. Interestingly, new evidence of a significant association with cancer risk has been described [10] and recent studies reassessed the association of K3326X *BRCA2* mutation with the risk of developing melanoma, urothelial, pancreatic, breast and ovarian cancers [11,12,13,14], while its role as modifier of genetic penetrance has been also reported [15]. By exploring the effect of this variant in our patient, we revealed clinical aspects apparently common to those previously described in patients with double *BRCA1*/*BRCA2* heterozygosity, including the earlier age of onset of cancer.

Therefore, we reviewed the literature with regard to double mutations on *BRCA1* and *BRCA2* genes in order to search for similarities in genotype–phenotype correlations and corresponding diagnostic and clinical implications.

## 2. Results

**Figure 1:**
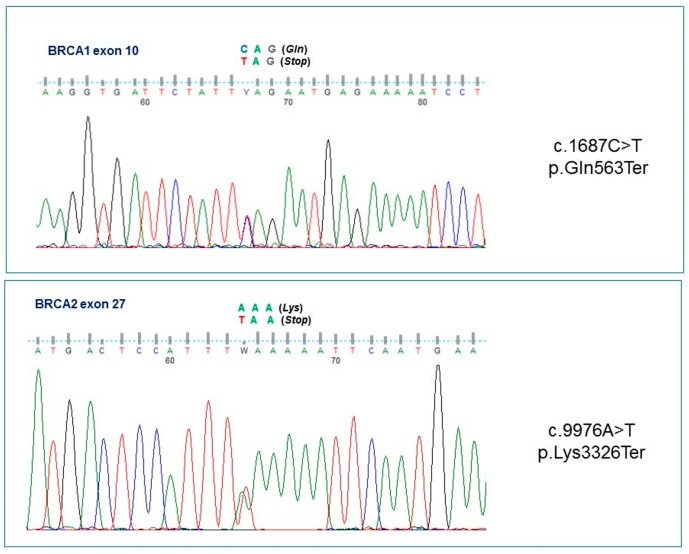
Direct sequence analysis of the *BRCA1* c.1687C>T and *BRCA2* c.9976A>T variants.

**Figure 2:**
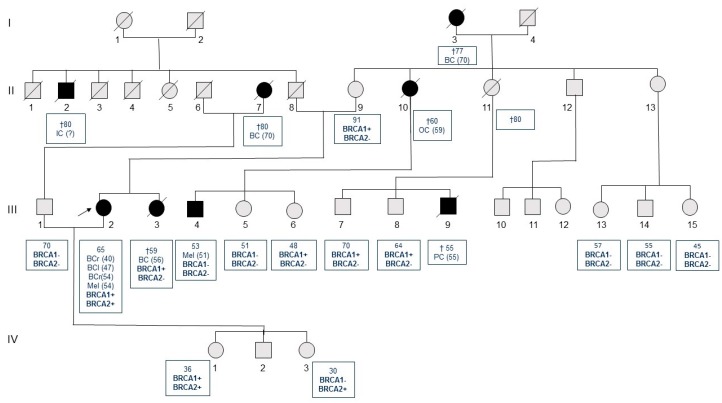
Pedigrees of the family with double hetyerozigosity for *BRCA1* c.1687C>T and *BRCA2* c.9976A>T variants. BC: breast cancer; BCr: breast cancer right; BCl: breast cancer left; OC: ovarian cancer; Mel: melanoma; PC: pancreatic cancer; IC: intestinal cancer. Black circles and squares: cancer patients; gray circles and squares: subjects without cancer; diagonal lines: dead subjects; †: age of death; arrow: proband.

Molecular analysis of the proband showed *BRCA1* c.1687C>T (p.Gln563Ter, rs80356898) and *BRCA2* c.9976A>T (p.Lys3326Ter; K3326*, rs11571833) heterozygous mutations bearing in both cases a premature stop codon in *BRCA1* at exon 10, and *BRCA2* at exon 27, respectively (Figure 1). The mutations recurred by genetic transmission to the patient’s asymptomatic daughters (Figure 2): the first, namely a 36-year-old (IV.1) inherited both *BRCA1* and *BRCA2* mutations; whereas the younger, aged 30 (IV.3) showed only the *BRCA2* variant. The proband’s asymptomatic 91-year-old mother (II.9) bore only the mutation in *BRCA1* in a similar fashion to the sister with breast cancer who died at 56 years of age (III.3), and for whom the analysis was performed on formalin-fixed, paraffin-embedded (FFPE) tissue. Mutational analysis, which turned out to be negative, was later extended to the proband’s 70-year-old husband, since he was reported as having a degree of relationship (first-degree cousins) with his mother (II.7), who died of breast cancer at the age of 70, and with the maternal grandmother of the proband (I.3), who died also at the age of 70 of breast cancer.

Genetic testing was also performed in other consenting relatives, and the following data refer to the presence of germinal *BRCA1* or *BRCA2* gene mutation. The mother’s sister had died at 60 years of age for a metastatic ovarian cancer (II.10) one year earlier. She had three children: a 53-year-old son (III.4), who had received a diagnosis of cutaneous melanoma two years before, but with no mutations; a 51-year-old daughter (III.5) carrying no mutations; and an asymptomatic 48-year-old daughter (III.6) positive to the mutation of the *BRCA1* gene. An additional sister of the mother’s proband (II.11) died at 80 years of age for causes unrelated to cancer, while her son died at 55 years of age of pancreatic cancer (III.9). Two other sons of 70 (III.7) and 64 (III.8) years of age were apparently asymptomatic, but both of them bore mutations in the *BRCA1* gene. Furthermore, three more cousins, daughters of the asymptomatic 80-year-old sister of the mother’s proband (II.13) of 57 (III.13), 55 (III.14) and 45 (III.15) years of age, were negative to the mutational analysis.

Since the *BRCA2* p.Lys3326* mutation has often been found in linkage disequilibrium with the presence of a pathogenic frameshift *BRCA2* c.6275_6276delTT (Leu2092ProfsTer7) and a non-pathogenic *BRCA2* c.9257-16T>C (IVS24-17T>C) mutation [16], we extended the analysis of both variants to all consenting subjects. None of the analyzed subjects presented these sequence variants. The sequence analysis performed on the proband paraffin-embedded melanoma samples revealed the presence of the two *BRCA1* and *BRCA2* variants and a novel *BRCA2* c.4297G>A somatic variant (p.Gly1433Arg). According to the data shown on the variant annotation file generated by analysis on Ion Reporter v.5.0 (Thermo Fisher Scientific Inc.), the somatic mutation *BRCA2* c.4297G>A has a PolyPhen score of 0.916 and a SIFT score of 0.22. Using the WEB-tool PolyPhen-2 (genetics.bwh.harvard.edu/pph2/) this mutation is predicted to be possibly damaging, with a score of 0.777 (sensitivity: 0.85; specifity: 0.92); while using another web tool SIFT Human Coding SNPs (http://sift.jcvi.org/www/SIFT_chr_coords_submit.html) this mutation is predicted to be tolerated, with a SIFT score of 0.23. This variant is classified in the COSMIC database (http://cancer.sanger.ac.uk/cosmic) (COSM5842418) and described with a functional analysis through hidden Markov models (FATHMM) prediction as pathogenic (score 0.72).

Finally, molecular screening of *CDKN2A*, *p14* (specific exon-1β) and *CDK4* genes, which are involved in approximately 20–40% of large, high-risk families, performed both on the proband and on the cousin affected by melanoma (III.4), did not identify any pathogenetic sequence variant.

We further completed an extensive review of world-wide literature using the PubMed database (https://www.ncbi.nlm.nih.gov/pubmed) and reviewing the references of retrieved articles. All studies involving Jewish Ashkenazi patients were deliberately excluded. In two papers we have amended the calculation of two patients (Table 1) [17,18,19]. From 1998 to 2017, 19 articles described 33 families with 54 subjects carrying a double *BRCA* mutation [2,8,9,17,19,20,21,22,23,24,25,26,27,28,29,30,31,32,33]. Table 1 summarizes the main data, including ethnicity, description of mutations, matrilineal or patrilineal transmission of variants, cancer type and age of onset, for a total of 56 cases (34 probands and 22 relatives), with the addition of our current report.

The types of mutations and combinations of mutations on *BRCA1* and *BRCA2* did not occur in any of the probands and/or families studied. In 18 cases out of 34, the paternal and maternal transmission is not determined. Analysis of data, documented with molecular analysis, shows that in 8 cases one of the parents transmitted both variants, in 3 cases both parents had a mutation, and in 2 cases a single variant was transmitted by one parent while the other one was wild-type. Finally, in 2 cases one of the mutations had been identified in one of the parents while the other had not been analyzed. The phenotypic expression in double heterozygosity varied from unilateral breast cancer at the age of 26, to asymptomatic at the age of 72. Forty-two cases (75%) had a primary cancer: 35 breast, 2 ovary, 2 prostate, and 1 case for cervix, caecum and stomach, respectively. Fourteen subjects (25.0%) were asymptomatic (ranging from 30 to 72 years of age), 8 of whom (14.2%) were older than 40. A secondary neoplasia was detected in 14 out of 42 patients.

Finally, the median age at diagnosis for breast cancer was 38 years (range 26 to 76), while the median age at diagnosis calculated for all primary cancers was 40 (range 26 to 76). In very few cases was it possible to compare the age at the onset of the first cancer of subjects carrying the double mutations, with their single heterozygous relatives. In particular, Heidemann et al., described six German non-Jewish subjects with double mutation in whom the onset of the first breast cancer was at 39.6 years mean age, compared to their single heterozygous female relative with a mean age of 52.4 years [32]. Expanding the analysis to the available literature on Caucasian double heterozygote (DH) patients, the authors confirmed the occurrence of the first cancer at a mean age of 41.4 years in patients with double mutation in contrast to 53.0 years in the relatives carrying a single *BRCA* mutation. The 95% confidence intervals between double heterozygotes and their single heterozygous relatives did not overlap [32].

## 3. Discussion

*BRCA1* c.1687C>T (p.Gln563Ter/Q563X, previously named 1806C>T) variant is a well-known and fully characterized mutation which is particularly common in European populations. In Austria and Slovenia, for example, it recurs approximately in 15% and 26% respectively of all *BRCA1* pathogenic variants, and haplotype analysis suggests a potential Austrian origin [34,35]. This mutation, related to lung, gastric and bilateral breast cancers, was found with remarkable frequency in Spain [36] and with a strong founder effect in hereditary ovarian/breast cancer Polish patients [37]. In 2003, Johannsson O.T. et al., linearized a breast lymph node metastasis from a 53-year-old Swedish woman carrying the Q563X mutation [38]. The histological analysis revealed a poorly differentiated adenocarcinoma, negative for estrogen receptor (ER), progesterone receptor (PgR), human epidermal growth factor receptor 2 (HER-2) and epidermal growth factor receptor (EGFR) [38]. A complete expression dataset is on the website Gene Expression Omnibus (https://www.ncbi.nlm.nih.gov/geo/query/acc.cgi?acc=GSM155216).

The *BRCA2* c.9976A>T (p.Lys3326*) variant was identified for the first time in 1996 during a mutational screening program completed on families with recurrent breast cancer in Nebraska. The study was carried out on 513 breast cancer patients and concluded that this mutation does not confer a highly penetrant susceptibility for inherited breast cancer [39]. Despite a number of observations supporting the theory of a close association between this variant and breast, pancreas, esophageal and squamous-cell lung cancers, parallel equally detailed studies supported the non-pathogenicity of this variant [12,16]. In particular, in a study of 95 families carrying the mutation, Higgs et al., showed that the previously reported associations with increased cancer risk are in many cases due to the concomitant presence of a pathogenic frameshift *BRCA2* mutation c.6275_6276delTT (Leu2092ProfsTer7) as well as to the non-pathogenic *BRCA2* c.9257-16T>C (IVS24-17T>C) that are often found in linkage disequilibrium with p.Lys3326* [16]. In 2015 Thompson et al., on the basis of a study performed on 2634 Australian breast and/or ovarian cancer patients, provided evidence on the association with a low to moderate increase of breast cancer risk [10]. More recently, a molecular screening of Swedish cutaneous malignant melanoma patients reported the presence of the p.Lys3326* mutation in 12 out of 452 unrelated patients, 8 of them with obvious familial transmission, and 4 with sporadic melanoma [11]. Finally, a further study performed on a Chinese population of 6064 patients and 8661 controls showed that *BRCA2* p.Lys3326* plays an important role in the genetic susceptibility to urinary tract cancers since it is apparently associated with increased risk of bladder cancer [13].

More recently, a massive epidemiological study evaluated the association between this *BRCA2* variant and the risk of breast, ovarian and prostate cancers. In this regard, researchers at the Collaborative Oncological Gene-Environment Study (COGS), analyzed data from 76,637 breast/prostate patients, including 7183 *BRCA1* and 5101 *BRCA2* mutation carriers in comparison with 83,796 control subjects. The study confirmed the association of the *BRCA2* K3326X variant with an increased risk of developing triple-negative breast and serous ovarian cancers irrespective of additional *BRCA2* pathogenic variants, while no association with prostate cancer risk was found [12]. Finally, in a study of 48 women with ovarian cancer and high risk of genetic inheritance but negative for both *BRCA1* and *BRCA2* mutations, Stafford et al., observed two patients carrying the K3326X variant, one with a concomitant *RAD51D* nonsense mutation and the other with an *ATM* frameshift mutation, having both developed breast and ovarian cancer [15]. Therefore, the authors argued that this variant is of minimal risk when inherited alone, but may acquire the function of strong modifier of penetrance when concomitant with a second *BRCA* pathogenic mutation [15].

In relation to the effect on cellular and biochemical properties of this variant in cancer cell lines, several complete studies have already been reported. In some cases the results show that the K3326X variants had no effect on the *BRCA2* function and indicate it as “neutral variant” [40,41]. However, a study performed in mouse cells deleted for the COOH terminus of *BRCA2* (amino acids 3140–3328) and irradiated with γ-radiation shows that the deletion of *BRCA2* could accelerate cell proliferation and stimulate cancer by defective MmRadSl-mediated DNA repair [42].

However, to date in the Breast Cancer Information Core database (https://research.nhgri.nih.gov/projects/bic/), the K3326X variant, which is found with a frequency of about 1% in the population, is still clinically classified in the “pending” category.

In our study, we have identified double heterozygosity in the proband and in her asymptomatic daughter. The asymptomatic mother of the proband has transmitted only the *BRCA1* mutation and, since the father’s sampling was not available, we cannot argue whether or not the *BRCA2* K3326X was due to a “de novo” variant, or to a patrilineal transmission. Unfortunately, all relatives of the father died without leaving children. The proband developed the first breast cancer at the age of 40, while the sister, with only the *BRCA1* mutation, had an age of diagnosis of 56 years. Furthermore, the maternal aunt and the maternal grandmother, both obligate carriers for the *BRCA1* variant, developed an ovarian cancer at the age of 59 and a breast cancer at 70, respectively.

From the overall examination of the mutational state and the pedigree, we have drawn some considerations. First, we observed that the proband and her sister, both suffering from ductal breast carcinoma, had a very different age of onset with a marked difference of about 16 years. Neither had preventive mastectomy or oophorectomy and the two patients lived in the same household with the same eating habits, environmental and lifestyle factors. The observation of all other relatives only carrying *BRCA1* mutations leads us to consider that the pathogenic variant p.Gln563Ter, despite bearing a premature stop codon at exon 10 in the *BRCA1* gene, is present with a low penetrance in this family. A paradigmatic example is offered by the mother of the two sisters (II9), a carrier of the mutation but not affected by any neoplasia despite her age of 90 years, and the maternal aunt (II10), an obligate carrier and with a diagnosis of ovarian cancer at 59 years of age. These differences cannot only be explained by anticipation, since the proband’s cousins, III6, III7 and III8, are also carriers of the *BRCA1* mutation but not affected by neoplasia at 48, 70 and 64 years, respectively (mean age 60.6 years).

We focused on the cancer’s earliest age of onset in the subject with double heterozygosity and a careful examination of literature revealed that this is in agreement with previous findings and was not unexpected in patients carrying double heterozygosity for *BRCA1* and *BRCA2* genes [32]. Similarly, a few published works also report earlier age of breast cancer onset in women with a double mutation for both *BRCA* genes while, to our knowledge, there are no similar data concerning a double mutation involving the K3326X *BRCA2* variant.

The first observations of a double mutation in the *BRCA1* and *BRCA2* genes was found in 1997 in a casistica of patients of Ashkenazi origin affected by breast and ovarian cancer carrying both the 185delAG in the *BRCA1* and the 6174delT *BRCA2* gene mutations [5,6]. In this population, three founder mutations, *BRCA1* 185delAG, *BRCA1* 5382insC and *BRCA2* 6174delT, are particularly common with an approximate frequency of 1%, 0.1%, and 1.4%, respectively [6,7,43]. Therefore, on the basis of predictive statistical calculations, the population carrier frequency of *BRCA1* and *BRCA2* mutations in the Ashkenazi population should be approximately 3%, with a presumable occurrence of double heterozygosity of 1 in 1800 individuals [8,9]. In a *BRCA1* and *BRCA2* mutational study performed on 10,000 subjects, including 3022 Ashkenazi, Frank et al., identified double mutations exclusively in the Ashkenazi population with a frequency of 11 of the 3022 tested individuals (0.36%), and 11 on 617 positive cases (1.8%) [44]. In a review of the literature on 34 Ashkenazi ancestry patients with double heterozygosis for *BRCA1* and *BRCA2*, Leegte et al., described no genotype–phenotype correlation between the double mutational status and the age of onset, the cumulative lifetime risks and the presence of multiple primary tumors [9]. In an additional Ashkenazi cohort of 1191 *BRCA* mutations, with carriers from 567 families, Lavie et al., identified 22 subjects (1.84%) from 17 families (2.9%) bearing the double *BRCA1*/*BRCA2* mutation [4]. However, all 22 subjects were females, 7 of whom had breast cancer (46%) and 1 had ovarian cancer (6.6%). The authors noted a younger age at the occurrence of cancer in patients carrying the double mutation (44.6 ± 13 years) with respect to patients with a single mutation (48.1 ± 13 years), deducing from the absence of other clinical differences among the two groups of patients that none of them would have benefited from dedicated diagnostic or therapeutic measures [4].

Conversely, in the non-Ashkenazi population the prevalence of *BRCA1* and *BRCA2* mutation carriers is approximately 0.24% and double heterozygosity is a rare phenomenon recurring in approximately 1 in 190,000 subjects [8,9,45]. However, data relative to carriers of the double mutation are available only in a very limited number of studies that usually describe only single case reports.

As accurately described in the results section, after a careful examination of the literature we have identified 19 articles describing 33 families with 54 subjects carrying a double *BRCA* mutation.

The examination of these data therefore suggests that the presence of the second K3326X variant in our case induces a phenotype characterized by early onset of the neoplasia in a manner similar to the other cases of double heterozygosity previously described. However, we must also keep in mind data from recent studies describing the mild association with the risk of developing breast and ovarian cancers [12] and assigning to this variant the role of a strong modifier of penetrance [15]. These findings should be considered during the counseling of carriers of a pathogenic mutation and a concomitant K3326X variant for a potentially earlier age of onset of the first cancer, as suggested also in other studies [32].

Finally, we would like to focus the melanoma found in our patient, in which next-generation sequencing (NGS) detected a somatic *BRCA2* variant c.4297G>A (p.Gly1433Arg) reported as pathogenic by the COSMIC database. As mentioned above, a recent study showed a significant association between the K3326X variant allele and melanoma [11]. Intriguingly, this missense mutation identified in our work has been previously reported as a somatic mutation in genetic screening performed on samples from patients affected by metastatic melanoma [46]. To date, the association between mutations of the *BRCA1*/*BRCA2* genes and the onset of melanoma is still being studied with the presence in literature of controversial or inconclusive evidence [47]. However, as suggested by other authors, patient carriers of a *BRCA1*/*BRCA2* mutations should be informed about the risk of skin cancer and be subjected to a periodic dermatological control [48].

We hope that our observations may add knowledge to this topic and contribute to further studies needed to verify these findings.

## 4. Materials and Methods

A 65-year-old woman was referred to our Oncogenomic Research Center for genetic counseling, including a pedigree analysis and a complete medical examination. The patient reported her personal and familial history of breast cancer since, at age of 40 years, she was diagnosed as having breast cancer on the left, and underwent a quadrantectomy. The pathologist described undifferentiated ductal, infiltrating carcinoma of the breast (G3—pT1N0M0), showing the estrogen receptor (ER) at 25% in tumor cells whereas the progesterone receptor (PgR), proliferation-related Ki-67 antigen (Ki-67) expression and HER-2 were undetectable. Seven years later, the patient developed a second ductal cancer still on the residual left breast with peculiar molecular aspects, namely triple-negative with a Ki-67 of 30%. At the age of 54, a further cancer on the right breast was diagnosed, and the patient underwent a mastectomy. The tumor was still triple negative, and showed Ki-67 expression on 48% of tumor cells. Together with the third breast cancer, the patient developed a pigmented flat cutaneous neoplasm of 1.1 cm in diameter, with irregular margins, that was surgically removed and described by the pathologist as melanoma in situ. A paraffin-embedded sample of the melanoma was recruited. Germline molecular analyses with sequencing of both *BRCA1* and *BRCA2* genes were completed.

Once mutational data were available, we extended the genetic screening to all family members to complete a co-segregational analysis (Figure 1). Written informed consent was obtained from each participating individual, and the study was performed under the Ethics Committee of the University of Bari approvals (number 5329, 5 July 2017) and in accordance with the principles embodied in the Declaration of Helsinki.

The patient’s sister also developed a breast cancer, histologically documented as a moderately differentiated ductal left breast carcinoma (G2—pT1Nsn0), with Ki67 at 60% and HER-2 at 90% on tumor cells, while both ER and PgR were not expressed.

Genomic DNA was isolated from the peripheral blood of the patient using the DNeasy^®^ blood and tissue Kit (QIAGEN Inc., Chatsworth, CA, USA) and quantified with a Qubit^®^3.0 fluorometer (Life Technologies™, Carlsbad, CA, USA). We employed 10 ng of DNA to prepare the barcoded library using the Ion AmpliSeq^TM^ Library kit 2.0 and the Ion Xpress^TM^ barcode adapters (Life Technologies™) on the Ion AmpliSeq™ *BRCA1* and *BRCA2* Panel (Life Technologies™) according to the manufacturer’s instructions. The library was purified with Agentcourt AMPure XP reagent (Beckman Coulter, Beverly, MA, USA) and quantified with the Ion Library Quantitation Kit (Life Technologies™) on the StepOne Plus system (Applied Biosystem, Foster City, CA 94404, USA). Template preparation was performed with the Ion OneTouch^TM^ 2 System (Life Technologies™) and Ion OneTouch^TM^ ES. Finally, sequencing was performed on a personal genome machine (PGM) using the Ion PGM^TM^ Hi-Q^TM^ View Sequencing kit (Life Technologies™) on the Ion 314 chip v2 set (Life Technologies™) at the 500 flows standard. The results of sequence analysis were analyzed using Torrent Suite Software 5.0.4 (Life Technologies™) aligning all reads to the human reference hg19 genome. Variant calling was performed running the Torrent Variant Caller plugin version 5.0.4.0. 

Specific sequencing reactions to confirm NGS data were performed using a Big Dye Terminator on a 3500 Series Genetic Analyzer (Thermo Fisher Scientific Inc., Foster City, CA 94404, USA). In order to exclude pre-analytical and analytical errors, PCR reactions and sequencing analyses were carried out on two different DNA extractions. For the control of both primer design and results, we referred to the ENSEMBL sequence *BRCA1* (ENST00000357654.7) and *BRCA2* (ENST00000380152.7).

We evaluated possible genetic factors contributing to the hereditary melanoma. For this reason, molecular screening for the *CDKN2A* with *p14* (specific exon-1β) and *CDK4* genes was performed both on the proband and the cousin (III.4) affected by melanoma, as previously described [49,50].

Each identified variant was investigated in its potential pathogenic role using prediction algorithms such as SIFT and Polyphen, and web databases such as the Breast Cancer Information Core (http://research.nhgri.nih.gov/bic/), COSMIC (http://cancer.sanger.ac.uk/cosmic), Leiden Open Variation Database (http://www.lovd.nl/3.0/home), dbSNP (http://www.ncbi.nlm.nih.gov/snp/), Ensembl (http://www.ensembl.org/index.html) and the Human Gene Mutation Database (http://www.hgmd.cf.ac.uk/ac/index.php).

## Figures and Tables

**Table 1:** Data of females with *BRCA1* and *BRCA2* double heterozygosity published in the literature. WT: Wild Type; ND: Not Determined; DH: double heterozygosity; breast u.: breast unilateral; breast b.: breast bilateral; ovarian b.: ovarian bilateral; Mel: melanoma.

Geographic Localization/Ethnic Group	*BRCA1* Mutation	*BRCA2* Mutation	Sex	Inheritance		Proband Cancer/Age of Onset (Years) [Relative with DH]	References
				Mother	Father		
Scottish	c.2389G>T	c.3067_3068insA	F	WT	ND	Breast 35	[20]
German descent	c.5080G>T	c.6405_6409delCTTAA.	M	*BRCA1*	ND	Asymptomatic 36 [Sister asymptomatic 34] [Brother asymptomatic 30]	[21]
Australia (no Jewish ancestry)	c.3769_3770delGA	c.5946_5946delT	F	WT	*BRCA2*	Breast < 40	[22]
Spain	c.5123C>A	c.6275_6276delTT	F	*BRCA1 BRCA2*	-	Breast 28 [Mother asymptomatic 70] [Sister asymptomatic 40] [Cousin asymptomatic 47] [Cousin asymptomatic 41] [Uncle prostate 66] [Aunt breast 70] [Aunt breast 66]	[23]
Korea	c.4981G>T	c.5946_5949delTGGA	F	*BRCA1 BRCA2*	-	Breast 33 [Mother stomach 62]	[17]
Korea	c.1516_1520del5	c.2798_2799delCA	F	ND	ND	Breast 26	
Korea	c.1656_1656delT	c.4599A>C	F	ND	ND	Breast 37	
Netherlands	c.2685_2686delAA	c.3487_3487delG	F	ND	ND	Ovarian 40, breast 45	[9]
Netherlands	c.2685_2686delAA	c.4449_4449delA	F	ND	ND	Breast 28	
European	c.962G>A	c.3170_3174delAGAAA	F	ND	ND	Breast 37	[2]
Italy	c.4285_4286insG	c.7738C>T	F	ND	ND	Breast 37	[24]
Australia	c.3331_3334delCAAG	c.631+2T>G	F	ND	ND	Breast 34, colon 35, breast 53 [Sister asymptomatic 65]	[25]
Italy	c.5263_5264insC	c.5796_5797delTA	F	*BRCA1*	*BRCA2*	Breast 38, ovarian 42	[26]
Italy	c.835_835delC	c.8195T>G	F	ND	ND	Breast 43	[27]
Italy	c.3916_3917delTT	c.5379_5379delG	F	WT	ND	Breast 30, ovarian 36	
Italy	c.1687C>T	c.6469C>T	F	ND	ND	Breast 46, ovarian 58	
Italy	c.2405_2406delTG	c.4284_4285insT	F	ND	ND	Breast and ovarian 52	
Denmark	c.5096G>A	c.631+4A>G	F	-	*BRCA1 BRCA2*	Breast 53, ovarian 59 [Father breast 76] [Son and daughter asymptomatic]	[28]
Caucasian	c.1961_1961delA	c.1444_1444delC	F	ND	ND	Ovarian b. 50	[29]
Caucasian (maternal Ashkenazi ancestry)	c.5266_5267insC	c.4829_4830delTG	F	ND	ND	Breast u. 40	
Korea	c.3627_3628insA	c.6724_6725delGA	F	ND	ND	Breast 26	[19]
Korea	c.390C>A	c.3018_3018delA	F	ND	ND	Breast 45	
Korea	c.5030_5033delCTAA	c.1399A>T	F	ND	ND	Breast 35	
Japan	c.188T>A	c.5576_5579delTTAA	F		*BRCA1 BRCA2*	Breast 55 [Father asymptomatic 51] [Cousin breast 41; Endometrial cancer 46]	[30]
Afrikaners	c.2635G>T	c.7934_7934delG	F	*BRCA1*	*BRCA2*	Breast 42 [Healthy second cousin 49]	[31]
Germany	c.5263_5264insC	c.5645C>A	F	WT	*BRCA1 BRCA2*	Breast b. 37; Ovarian b. 63 [Father prostate 68]	[32]
Germany	c.66_67delAG	c.5722_5723delCT	F	*BRCA1*	*BRCA2*	Breast u. 32	
Germany	c.962G>A	c.2231C>G	F	*BRCA1 BRCA2*	WT	Breast b. 31, 35 [Mother breast 40]	
Germany	c.3910_3910delG	c.2830A>T	F	*BRCA1 BRCA2*	WT	Breast u. 39 [Mother breast 34; another cancer not reported 35]	
Germany	c.5193+1_5193+1delG	c.658_659delGT	F	ND	ND	Coecum 58, ovarian 61	
Germany	c.3700_3704delGTAAA	c.1813_1814insA	F	ND	ND	Cervix 26, breast 40	
Italy	c.547+2T>A	c.2830A>T c.426-57A>G	F	-	*BRCA1 BRCA2*	Breast 35 [Father asymptomatic 72]	[33]
France	c.1016_1017insA	c.6814_6814delA	F	*BRCA1*	WT	Breast 46	[8]
Italy	c.1687C>T	c.9976A>T	F	*BRCA1*	ND	Breast u (40), breast u (47), breast b (54), Mel (54) [Asymptomatic daughter (36)]	This report
